# Cancer Surveillance Guideline for individuals with *PTEN* hamartoma tumour syndrome

**DOI:** 10.1038/s41431-020-0651-7

**Published:** 2020-06-12

**Authors:** Marc Tischkowitz, Chrystelle Colas, Sjaak Pouwels, Nicoline Hoogerbrugge, PHTS Guideline Development Group, Tanya Bisseling, Virginie Bubien, Frederic Caux, Nathalie Chabbert-Buffet, Chrystelle Colas, Sophie Da Mota Gomes, Martin Gotthardt, Nicoline Hoogerbrugge, Marleen Kets, Katherine L. Lachlan, Thera P. Links, Michel Longy, Ritse Mann, Sjaak Pouwels, Leo Schultze Kool, Robert K. Semple, Ian Stock, Marc Tischkowitz, Janet Vos, The European Reference Network GENTURIS, Nicoline Hoogerbrugge, Marjolijn Ligtenberg, Rianne Oostenbrink, Rolf Sijmons, Gareth Evans, Emma Woodward, Marc Tischkowitz, Eamonn Maher, Rosalie E. Ferner, Stefan Aretz, Isabel Spier, Verena Steinke-Lange, Elke Holinski-Feder, Evelin Schröck, Thierry Frebourg, Claude Houdayer, Chrystelle Colas, Pierre Wolkenstein, Vincent Bours, Eric Legius, Bruce Poppe, Kathleen Claes, Robin de Putter, Ignacio Blanco Guillermo, Gabriel Capella, Joan Brunet Vidal, Conxi Lázaro, Judith Balmaña, Hector Salvador Hernandez, Carla Oliveira, Manuel Teixeira, Svetlana Bajalica-Lagercrantz, Emma Tham, Jan Lubinski, Karolina Ertmanska, Bela Melegh, Mateja Krajc, Ana Blatnik, Sirkku Peltonen, Marja Hietala

**Affiliations:** 1 Department of Medical Genetics, National Institute for Health Research Cambridge Biomedical Research Centre, Cambridge University Hospital NHS Foundation Trust, Cambridge, UK; 2 Department of Genetics, Institut Curie, Paris Sciences et Lettres Research University, Paris, France; 3 Haaglanden Medical Center, The Hague, The Netherlands; 4 Department of Human Genetics, Radboud University Medical Center, Nijmegen, The Netherlands; 5 Department of Gastroenterology and Hepatology, Radboud University Medical Center, Nijmegen, The Netherlands; 6 Cancer Genetics Unit, Institut Bergonié, Bordeaux, France; 7 Dermatology, Avicenne Hospital, Assistance Publique-Hôpitaux de Paris, Université Paris 13, Bobigny, France; 8 Department of Obstetrics and Gynaecology, Tenon University Hospital, Assistance Publique des Hôpitaux de Paris, Sorbonne University, Paris, France; 9 Patient Representative, Paris, France; 10 Department of Radiology and Nuclear Medicine, Radboud University Medical Center, Nijmegen, The Netherlands; 11 Family Cancer Clinic, The Netherlands Cancer Institute-Antoni van Leeuwenhoek Hospital, Amsterdam, The Netherlands; 12 Wessex Clinical Genetics Service, University Hospitals Southampton NHS Trust, Southampton, UK; 13 Endocrinology, University Medical Centre Groningen, Groningen, The Netherlands; 14 Centre for Cardiovascular Science, Queen’s Medical Research Institute, University of Edinburgh, Edinburgh, UK; 15 Patient Representative, PTEN UK and Ireland Patient Group (PTENUKI), London, UK; 16 Erasmus Medical Center, Rotterdam, The Netherlands; 17 University Medical Center, Groningen, The Netherlands; 18 Genomic Medicine, Central Manchester Foundation Trust, Manchester, UK; 19 Guy’s and St. Thomas’ NHS Foundation Trust, London, UK; 20 University Hospital Bonn, Bonn, Germany; 21 Medizinisch Genetisches Zentrum, Munich, Germany; 22 Hereditary Cancer Syndrome Center Dresden, Dresden, Germany; 23 Rouen University Hospital, Rouen, France; 24 University Hospital Henri Mondor-National Referral Center, Créteil, France; 25 University Hospital, Liege, Belgium; 26 University Hospital Leuven, Leuven, Belgium; 27 Ghent University Hospital, Ghent, Belgium; 28 Hospital Universitari Germans Trias i Pujol y ICO Badalona, lnstitut Catala d’Oncologia, Barcelona, Spain; 29 Hospital Sant Joan de Déu, Barcelona, Spain; 30 Porto Comprehensive Cancer Center, Porto, Portugal; 31 Karolinska University Hospital, Stockholm, Sweden; 32 Pomeranian Medical University - University Clinical Hospital n1, Szczecin, Poland; 33 University of Pécs, Pécs, Hungary; 34 Institute of Oncology, Ljubljana, Slovenia; 35 Turku University Hospital, Turku, Finland

**Keywords:** Preventive medicine, Cancer genetics

## Abstract

*PTEN* hamartoma tumour syndrome is a diverse multi-system disorder predisposing to the development of hamartomatous growths, increasing risk of breast, thyroid, renal cancer, and possibly increasing risk of endometrial cancer, colorectal cancer and melanoma. There is no international consensus on cancer surveillance in PHTS and all current guidelines are based on expert opinion. A comprehensive literature review was undertaken and guidelines were developed by clinicians with expertise from clinical genetics, gynaecology, endocrinology, dermatology, radiology, gastroenterology and general surgery, together with affected individuals and their representatives. Recommendations were put forward for surveillance for breast, thyroid and renal cancers. Limited recommendations were developed for other sites including endometrial, colon and skin. The proposed cancer surveillance recommendations for PHTS require a coordinated multidisciplinary approach and significant patient commitment. The evidence base for cancer surveillance in this guideline are limited, emphasising the need for prospective evaluation of the effectiveness of surveillance in the PHTS population.

## Introduction

*PTEN* hamartoma tumour syndrome (PHTS), OMIM 158350, ORPHA:306498, is caused by germline variants that affect function of the *PTEN* (phosphatase and tensin homologue) gene, henceforth called “pathogenic variants” (PV). It is a diverse multi-system disorder that encompasses Cowden syndrome, Bannayan–Riley–Ruvalcaba syndrome and Proteus-like syndrome, individuals with PHTS are at increased risk of breast, thyroid, renal cancer, and possibly endometrial cancer, colorectal cancer and melanoma [1].

The projected estimated lifetime risks of cancer in individuals with PHTS range from 85 to 89% for any cancer, 67 to 85% for female breast cancer, 6 to 38% for thyroid cancer, 2 to 28% for endometrial cancer, 2 to 34% for renal cancer, 9 to 20% for colorectal cancer and 0 to 6% for melanoma [2–6]. These estimates and those given in Table 1 are likely to be at the upper end of the true range because of likely ascertainment bias in studies published to date. Moreover these estimates are projections based on small datasets and have wide confidence intervals. Ultimately, larger prospective longitudinal studies, including those individuals diagnosed in childhood because of developmental problems, and asymptomatic relatives with *PTEN* PVs, will be needed to define the risk more accurately.

**Table 1:** Estimates for projected lifetime risks of tumours in individuals with PHTS.

Cancer	Current risk estimates	Publications
Breast	Cancer—lifetime up to 85% Median age at diagnosis 38–46 years	81% [2], 85% [6], 77% [5]
Thyroid	Cancer—lifetime 35% Median age at diagnosis 37 years Up to 75% risk of multinodular goitre, adenomatous nodules and follicular adenomas	21% [2], 35% [6], 38% [5]
Endometrial	Cancer—lifetime up to 28% Risk starts late 30 s–early 40 s	19% [2], 28% [6], 2% [5]
Renal	>Cancer—lifetime up to 34% (mostly papillary) Risk starts late 40 s	15% [2], 34% [6], 2% [5]
Colorectal	Cancer—lifetime up to 16%; Risk starts late 30 s More than 90% have polyps, which may be symptomatic	16% [2], 9%, [6] 3% [5], 13% [7]
Skin and vascular system	Melanoma—~5% Many non-malignant lesions	6% [6]

PHTS is rare and its clinical diagnosis relies on characteristic signs and symptoms with variable expressivity, followed by confirmatory genetic testing. Early identification of affected individuals, which often precedes development of advanced cancer by several years, allows appropriate surveillance to be instituted, which is key to timely detection of lesions. Genotype–phenotype analysis has not been conclusive. A single study by Tan et al. found a correlation between promoter PVs and breast cancer and between nonsense PVs and colorectal cancer, this remains unconfirmed [6]. There are thus currently no specific PVs established that help to stratify patients for surveillance.

Diagnostic criteria for PHTS have been published and are regularly updated by the National Comprehensive Cancer Network^®^ (National Comprehensive Cancer Network^®^ [NCCN^®^], 2019). They are divided into major and minor criteria, and various combinations can be used to reach a diagnosis. The availability of these criteria aids clinicians in achieving consistency in clinical case definition. Conversely, gene-specific criteria for the interpretation of *PTEN* variants have been developed by the ClinGen *PTEN* Expert Panel [7]. They offer a more bespoke approach to the American College of Medical Genetics variant interpretation guidelines and are a helpful tool for those involved in *PTEN* variant classification. In this light it is important to keep in mind that historic reports did not interpret variants with the same stringency as is now applied.

Individuals with PHTS are at risk of several different cancers which are amenable to early detection, but surveillance protocols are complex, and there are no data available documenting a consequent reduction in morbidity and mortality, nor evaluating how well surveillance is coordinated across countries. Moreover, there is no international consensus on cancer surveillance in PHTS and all current guidelines are based on expert opinion. Guidelines have been published by the National Comprehensive Cancer Network® (NCCN®), and in the United Kingdom guidelines have been developed for use in the National Health Service by the UK Cancer Genetics Group. Here we propose guidelines for member states of the European Union which could also be used by other countries.

## Scope of the guidelines

This guideline is intended to address cancer surveillance of individuals with PHTS and has been elaborated by members of the European Reference Network (ERN) for Genetic Tumour Risk Syndromes (GENTURIS). It aims specifically to integrate available information to assist healthcare professionals in evidence-based surveillance of individuals with a confirmed germline pathogenic variant in *PTEN*. It addresses surveillance for increased risk of cancer tailored to tumour site, offers guidance on the imaging modality that should be used for surveillance, on the age at which to start surveillance for each cancer, and on frequency of subsequent surveillance. The scope of this guideline was set to determine what is currently known about the efficacy, frequency and potential methods for surveillance, for breast, thyroid, renal, endometrial or colorectal cancers in PHTS. For melanoma, the risk is not sufficiently established to consider additional surveillance at present. There is clearly an increased risk of cancers in PHTS and this guideline seeks to clarify this risk, and to suggest an approach to screening that pragmatically balances the risk of harm from the over-diagnosis of cancer with the potential benefits of early identification of cancers, based on current incomplete evidence.

## Methods

ERN Guidelines on Cancer Surveillance Guideline for Individuals PHTS consists of clinicians with expertise from clinical genetics, gynaecology, endocrinology, dermatology, radiology, gastroenterology, general surgery and affected individuals and their representatives. The Guideline Development Group was led by a Core Writing Group of ERN GENTURIS HCP Members from different Member States and who are recognised experts in specialised clinical practice in the diagnosis and management of PHTS. The Core Writing Group leads had joint meetings with a Patient Advisory Group composed of affected individuals and parent representatives that have experience with PHTS syndrome.

The elaboration of these guidelines then additionally involved external experts from different speciality areas relevant to the scope of the guideline.

The guidelines were developed on the basis of 131 published articles extracted from Pubmed, using the following terms: (screening [title/abstract] OR surveillance [title/abstract]) AND (*PTEN* [title] OR Cowden [Title]) AND “humans” [MeSH Terms].

Additional papers were requested from experts in the field and references of all the papers were considered. Papers were included if they contained any data on screening or surveillance and renal cell, thyroid, endometrial, breast or colorectal cancer in PHTS.

As is typical for many rare diseases, the volume of peer-reviewed evidence available to consider for these guidelines was small and came from a limited number of articles, which typically reported on small samples or series. To balance the weight of both published evidence and quantify the wealth of expert experience and knowledge, we have used for evidence grading the following scale: (i) *strong evidence*: consistent evidence and new evidence unlikely to change recommendation and expert consensus; (ii) *moderate evidence*: expert consensus or majority decision but with inconsistent evidence or significant new evidence expected and (iii) *weak evidence*: inconsistent evidence AND limited expert agreement.

## Recommendations

The agreed recommendations are summarised in Table 2.

**Table 2:** Guideline summary: cancer surveillance protocol for individuals with *PTEN* hamartoma tumour syndrome.

	Surveillance	Interval	From age	Evidence
Breast cancer	MRI	Yearly	30	Strong
	Mammography	Every 2 years	40	Moderate
	Risk-reducing surgery offered	–	–	Moderate
Thyroid cancer	Ultrasound	Yearly	18^a^	Strong
Renal cancer	Ultrasound	Every 2 years	40	Moderate
Colorectal cancer	Baseline colonoscopy^b^	–	35–40	Moderate
Melanoma	Baseline skin examination^c^	–	30	Weak
Endometrial cancer	Not recommended^d^	–	–	Weak

^a^Moderate evidence for age of commencement of surveillance.

^b^Consider further surveillance as required by the gastroenterologist.

^c^Consider further surveillance as required by the dermatologist.

^d^Consider surveillance as part of clinical trial.

### Breast

There is strong evidence of an increased risk of breast cancer in women with germline PVs in *PTEN* [3, 5, 6]. However, there was weak evidence to address the question of which modality should be used for surveillance and how surveillance impacts on morbidity and mortality in PHTS. Published studies to date suggest that the breast cancer risk in PHTS is similar to that in women with germline PVs in *BRCA1/BRCA2*. Therefore, many of the recommendations are derived from the much larger evidence base which exists for those hereditary breast cancer predisposition syndromes. For those centres that wish to use mammography there is no evidence of additional incremental benefit in performing mammography more frequently than every 2 years with surveillance in the intervening years being better performed by magnetic resonance imaging (MRI).

**Table:** 

Breast		
No	Recommendations	Grading
1	Women *should* be screened for breast cancer	Strong
2	Surveillance for breast cancer in PHTS *should* use MRI (MRI should be ideally conducted between day 5 and day 12 of the menstrual cycle)	Strong
3	Surveillance for breast cancer with MRI *should probably* start at 30	Strong
4	Women *should* be screened for breast cancer annually	Strong
5.	If surveillance for breast cancer in PHTS additionally includes mammography this *should* be undertaken no more frequently than every 2 years	Moderate
6	If surveillance for breast cancer with mammography is offered this *should probably* start at 40	Moderate
7	Risk reduction surgery *should* be offered using the same considerations as for women with germline *BRCA1/BRCA2* pathogenic variants	Moderate

### Thyroid

There is strong evidence of an increased risk of thyroid carcinoma in PHTS with evidence that this can arise at relatively young ages [2, 3, 5, 6, 8, 9]. However, no study to date has investigated which modality should be used for surveillance or how surveillance impacts on morbidity and mortality in PHTS. Although there are occasional reported cases of children with PHTS developing thyroid carcinoma [8, 9] the evidence is weak and does not support this being frequent enough to justify the significant additional burden that would be required to screen all individuals throughout childhood. There is strong evidence that identification of early stage thyroid carcinomas in other populations leads to better outcomes [2] and that ultrasound is an appropriate modality for surveillance for thyroid carcinomas.

**Table:** 

Thyroid		
No	Recommendations	Grading
1	Individuals *should* be offered surveillance for thyroid cancer	Strong
2	Surveillance for thyroid cancer in PHTS *should* be by US	Strong
3	Surveillance for thyroid cancer *should probably* start at 18 years	Moderate
4	Individuals *should probably* be offered surveillance for thyroid cancer annually	Moderate

### Kidney

There is strong evidence of an increased risk of renal cell carcinoma (RCC) in individuals with PHTS. However, no study to date has investigated which modality should be used for surveillance and how surveillance impacts on morbidity and mortality in PHTS. One study of 219 individuals with PHTS identified nine individuals with prevalent or incident history of RCC [10]. Histopathological review of eight of these revealed complex tumours with mixed cell types including papillary and chromophobe. There is strong evidence that identification of early stage RCCs in other populations leads to significantly better outcomes [11]. There is strong evidence, in other populations that ultrasound is an appropriate modality for surveillance for RCCs [12, 13]. It is possible that ultrasonography will miss more aggressive tumours seen in some predisposition syndromes such as Hereditary leiomyomatosis and renal cell cancer where surveillance with renal MRI is advocated [14], but at present there are insufficient data to recommend renal MRI in PHTS.

**Table:** 

Kidney		
No	Recommendations	Grading
1	Individuals *should* be offered surveillance for renal cell carcinoma (RCC).	*Moderate*
2	Surveillance for RCC in PHTS *should* be by ultrasound	*Moderate*
3	Surveillance for RCC *should probably* start at 40.	*Moderate*
4	Surveillance for RCC *should probably* be at least every 2 years.	Moderate

### Colon

Polyps are common in PHTS, and these are typically hamartomas, although other types can also occur [15, 16] There is weak evidence regarding colorectal cancer risk in PHTS with some studies observing a modest increased risk estimated to be 9–16% [3, 6, 15, 16], but this is not a consistent finding [5]. In the studies that showed an association the mean age at diagnosis of colorectal cancer varied from 44 to 58 years. Therefore, the recommendations for surveillance are broadly those that apply to the general population, with the addition of a baseline colonoscopy undertaken at 35–40 to assess polyp load. Further surveillance would be determined by the findings at baseline colonoscopy; if this was normal (no polyps) then general population screening guidelines should be followed.

**Table:** 

Colon		
No	Recommendations	Grading
1	Baseline colonoscopy should be undertaken at 35–40 years to assess polyp load.	Moderate
2	If the baseline colonoscopy is normal, individuals *probably should not* be screened for colorectal cancer at any greater frequency or earlier age than the general population.	Moderate

### Skin

There is weak evidence regarding skin cancer risk in *PHTS*. Therefore, the recommendations for surveillance should be those that apply to the general population, with the addition of a baseline skin examination at 30 by a dermatologist who can determine whether further surveillance is required and whether this should be done by a specialist or generalist.

**Table:** 

Skin		
No	Recommendations	Grading
1	Individuals *probably should* have a baseline skin examination at age 30, further surveillance as required (consider every 2 years).	Weak

### Endometrial

There is weak evidence regarding endometrial cancer risk in *PHTS*. The limited evidence suggests that if these cancers occur, they behave similarly to endometrial cancers in other cancer syndromes. If surveillance for endometrial cancer is offered it should be as part of a clinical trial. Women should be advised to report red flag symptoms (e.g. post menopausal or irregular vaginal bleeding) without delay so they are promptly investigated.

**Table:** 

Endometrial		
No	Recommendations	Grading
1	Women *should probably not* be screened for endometrial cancer.	*Moderate*
2^a^	If surveillance for endometrial cancer is offered it *should* be as part of a clinical trial.	*Strong*
3^a^	If surveillance for endometrial cancer is offered, it *should probably* start at 40.	*Weak*
4^a^	If surveillance for endometrial cancer is offered, it should probably be done at least annually.	*Weak*
5	There is no clinical indication for endometrial cancer risk reduction surgery (hysterectomy).	Weak

^a^NB: Recommendations 2–5, should be undertaken as part of a clinical trial.

## Discussion

The goal of cancer surveillance is to detect cancer at an earlier stage than symptomatic presentation, when interventions have a better chance of being curative. The proposed surveillance recommendations for PHTS require a coordinated multidisciplinary approach and significant patient commitment. It is also important to remember that individuals with PHTS are at risk of multiple cancers over their lifetime and surveillance for second cancers should not be overlooked. As this is a very rare condition there is unlikely to be a large health economic burden for the health service if these guidelines are implemented. However, surveillance in each individual is complex and additional resources may need to be put in place for those health service providers that are planning to offer surveillance at a local and regional level. For this reason, we recommend that individuals who are at 50% risk of a *PTEN* PV initially proceed with genetic testing to determine whether or not they require surveillance. For individuals that meet the diagnostic criteria for PHTS, but where no PV has been identified, surveillance should be tailored on a case by case base, taking into account the personal and family history of cancer. PHTS-related cancers are predominately adult onset and no specific recommendations have been made for non-malignant manifestations in adults or for the paediatric PHTS population whose management has been addressed elsewhere [17].

The evidence base for cancer surveillance in this guideline are limited. The quality of the evidence regarding baseline risk has been rated as weak as it is non-randomised and based on small numbers. A better understanding of the age-related penetrance and the extent of the risk increase of cancer is critical to improve risk counselling and risk-based recommendations for cancer prevention and treatment. We therefore recommend that national and international registries are established to collect prospective data on PHTS individuals undergoing surveillance.

Research should focus on understanding factors affecting the risk of each type of cancer and translate this into more accurate and personalised cancer risk estimates. There are no data regarding preventative drugs (e.g. tamoxifen for breast cancer, aspirin for bowel polyps/cancer) in PHTS. Furthermore, research is needed to gain insights into the cancer treatment and prognosis of PHTS patients. At present cancer treatment of PHTS patients is similar to that for sporadic cancers. Understanding the relation between patient, tumour and treatment characteristics would be the first step towards developing a tailored treatment for PHTS patients. As PHTS is a rare disease, collaboration supported by a common/central PHTS registry infrastructure is essential to underpin this. In addition, the role of prophylactic surgery has not been evaluated for this syndrome and requires further research.

Patient education is a critical component of effective cancer surveillance. This relates to both prevention (healthy living and avoidance of cancer-causing behaviours) and early detection (awareness of red flay symptoms for the key cancers). Patient information groups can assist greatly with these aspects and are a powerful resource for individuals with PHTS.

Early detection and surveillance of hereditary cancers relies on established imaging methods such as ultrasonography and MRI. It is imperative that new surveillance techniques are developed, that are not only more specific in their detection ability, but also more easily available and affordable for the healthcare systems. Utilisation of non-invasive “liquid biopsy” technologies able to identify the presence of genetic material from cancer cells in the blood or molecular markers in urine or saliva that can identify precursor lesions or cancer at its earliest stages are still being evaluated in a research setting and individuals with PHTS would be a good target population to trial these. Another area of need is the identification and validation of biomarkers that may distinguish aggressive, life-threatening cancers from more indolent types. Above all, it will be important to prospectively evaluate the effectiveness of surveillance in the PHTS population and to foster global collaborations with data sharing to enhance clinical care and research opportunities for this group of high-risk individuals.

### Website

The complete guidelines can be downloaded from the ERN website: https://www.genturis.eu.
